# Assessment of Lacrimal Duct Patency in Patients Undergoing Endoscopic Medial Maxillectomy

**DOI:** 10.3390/jcm10020245

**Published:** 2021-01-12

**Authors:** Andrzej Sieśkiewicz, Tomasz Łysoń, Marek Rogowski, Marek Bielecki, Ewa Gindzienska-Sieskiewicz, Ewa Olszewska, Pawel Bielecki

**Affiliations:** 1 Department of Otolaryngology, Medical University of Bialystok, 15-276 Bialystok, Poland; erogowski@onet.eu (M.R.); ewaolsz@yahoo.com (E.O.); pbieleckimd@gmail.com (P.B.); 2 Department of Neurosurgery, Medical University of Bialystok, 15-276 Bialystok, Poland; nch@umb.edu.pl; 3 Department of Orthopedics and Traumatology, Medical University of Bialystok, 15-276 Bialystok, Poland; ortopamb@o2.pl; 4 Department of Rheumatology and Internal Diseases, Medical University of Bialystok, 15-276 Bialystok, Poland; reum@umb.edu.pl

**Keywords:** epiphora, medial maxillectomy, lacrimal duct

## Abstract

Purpose: The risk of epiphora after medial maxillectomy with lacrimal duct transection is difficult to assess. The data available in the literature are inconclusive due to various operating techniques used by the authors of medical publications, different additional procedures aimed at improving tear drainage after maxillectomy, and a variety of lacrimal duct patency assessment techniques. The aim of our work was to assess the anatomical and functional patency of lacrimal ducts after medial maxillectomy without performing additional procedures to improve tear drainage as well as comparison of the results obtained with different assessment tests. Materials and methods: 21 patients who underwent medial maxillectomy in the years 2016–2019 were assessed for discomfort and epiphora based on patients’ own reports and basic clinical examination, lacrimal duct rinse test, the Munk score, and a modified endoscopic Jones I test. Results: Gradually increasing the sensitivity of the assessment method resulted in an increase in the number of patients with potential tear drainage disorders, starting from 0% in the rinsing test, 4.8% self-reported tearing complaints, 14.3% Munk score, and 19% modified endoscopic Jones I test. Conclusions: The study results revealed that a small fraction of patients tend to report epiphora as a consequence of medial maxillectomy themselves. Subtle functional disorders, which are not particularly bothersome to patients, are more common. More sensitive lacrimal duct patency tests reveal more cases of tear drainage disorders. The results of studies assessing the incidence of epiphora after medial maxillectomy appear to depend on the type of test used.

## 1. Introduction

The technological development of visualisation techniques in recent years has resulted in endoscopic transnasal surgeries becoming the most frequently performed ENT procedures. In the case of surgeries performed in the region of the maxillary sinus, due to the anatomical proximity of this sinus and nasolacrimal duct (which drains tears from the lacrimal sac to the inferior nasal meatus), it is particularly susceptible to iatrogenic injury [1,2]. Bogler et al. [1], based on intraoperative fluorescein tests performed immediately after ethmoidectomy and infundibullotomy, confirmed lacrimal duct damage in 15% of the operated patients. Despite relatively frequent intraoperative lacrimal duct injury, symptoms of postoperative lacrimal duct obstruction occur only in 0.3–1.7% of patients [2,3,4].

Contrary to the standard functional endoscopic surgery of the maxillary and ethmoid sinuses, which aims to precisely widen the natural opening of the sinus and to avoid damage to the nasolacrimal duct, in endoscopic surgeries of tumours located in this area, it is sometimes necessary to remove the entire medial wall of the maxillary sinus together with the nasolacrimal duct. An example of this type of procedure is endoscopic medial maxillectomy (EMM) performed to gain better access to the lesions located in the antero-inferior portion of the maxillary sinus [2,5]. Transecting the nasolacrimal duct during this type of surgery carries a potential risk of lacrimal duct obstruction postoperatively. Significantly, this risk is difficult to assess, as the data available in the literature are inconclusive due to the different operating techniques used by the authors of medical publications; various additional procedures aimed at improving tear drainage, which were carried out during maxillectomy; and a variety of methods, which do not always prove to be objective, used to assess tear patency following the surgery.

The aim of our study was to assess the anatomical and functional patency of lacrimal ducts in patients who underwent medial maxillectomy with transection of the nasolacrimal duct at the level of inferior orbital wall without performing additional procedures to improve tear drainage as well as comparison of the results obtained with different assessment tests.

## 2. Experimental Section

Materials and Methods

**Figure 1:**
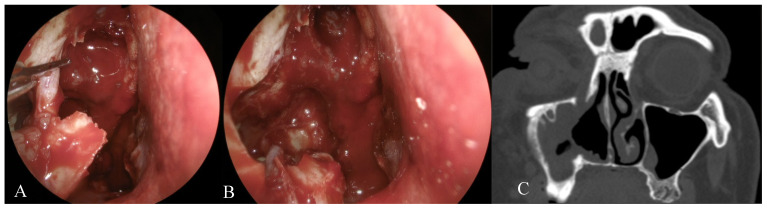
Transection of nasolacrimal duct at the level of orbital floor (**A**). Nasolacrimal duct removed, no additional procedures aimed at improving tear drainage were performed (**B**). Postoperative CT of the same patient—amputated nasolacrimal duct on the right side (**C**).

The study included 21 patients who underwent medial maxillectomy for inverted papilloma (17 patients), squamous cell carcinoma (3 patients) and esthesioneuroblastoma (1 patient) in the years 2016–2019. During the procedure, the nasolacrimal duct was transected at the level of lower orbital wall, i.e., at the junction of the lacrimal sac with nasolacrimal duct. No additional procedures were used to improve lacrimal duct patency either during the operating procedure or in the postoperative period (Figure 1).

Discomfort and tearing were assessed at least 6 months (between 6 and 44 months) following the surgery, based on patients’ own reports and basic clinical examination, lacrimal duct rinse test, the Munk score [6], and a modified endoscopic Jones I test with fluorescein dye [7].

During the clinical examination, probing of lacrimal pathway through the inferior lacrimal punctum was performed, and the patients were interviewed for complaints related to tear drainage. Attention was paid to spontaneous eye tearing and tear retention symptoms.

The saline rinsing test was performed using a cannula inserted through the inferior lacrimal point into the inferior canaliculus. Following this, endoscopic observation of fluid outflow in the nasal meatus (anatomical patency) was performed.

The Munk score [6] was used to determine the intensity of epiphora (Munk score: 0, no epiphora; 1, occasional epiphora requiring wiping less than twice a day; 2, wiping 2–4 times per day; 3, wiping 5–10 times per day; and 4, wiping more than 10 times per day or continuous tearing).

A modified Jones I test combined with endoscopic observation was used to assess functional lacrimal duct patency. In this test, two drops of 2% fluorescein were instilled into the conjunctival fornix and subsequently endoscopic observation of dye traces in the region of the new ostium of the lacrimal pathway in the nasal meatus after 1, 3 and 5 min was performed.

## 3. Results

Following surgical treatment, only 1 out of 21 patients (4.8%) reported epiphora on the operated side in the initial clinical evaluation. None of the patients reported inflammation of the lacrimal sac during the follow-up period.

The rinse test and probing confirmed anatomical patency in all patients—outflow of the saline solution from the amputated nasolacrimal duct was observed endoscopically.

Munk’s grade 2 of epiphora was noted in 1 patient (4.8%), grade 1 in 2 patients (9.5%) and grade 0 (no eye tearing) in the remaining 18 patients.

**Figure 2:**
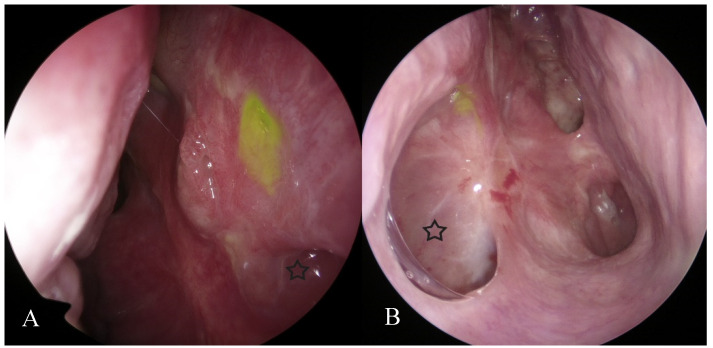
Modified endoscopic Jones I test. Profuse leak of floresceine dye could be observed almost immediately (within first minute) after application in most of our patients (**A**) and weak leak observed after more than 3 min after dye application in patients with tear drainage disorders (**B**), asterix—maxillary sinus.

The functional patency test showed a significant increase (over 3 min) in the time required for fluorescein dye to reach the nasal meatus in four patients (19%). In all these patients, the intensity of fluorescein leakage, as assessed by the surgeon, was conspicuously lower than in the remaining study group patients (Figure 2).

## 4. Discussion

The results of our study show that persistent epiphora after medial maxillectomy with nasolacrimal duct transection is relatively rare. Subtle functional disorders, which are not particularly bothersome to the patients and often neglected by them, are more frequent.

The results of assessment of incidence of epiphora following medial maxillectomy appear to depend largely on the type of test used.

Only one patient in the study group (4.8%) reported eye tearing discomfort following the surgery. At the same time, a clinical examination with lacrimal duct probing and flushing did not reveal any patency disorders in any of the operated patients. Situations in which patients report watering of the eyes while the anatomical patency assessed by the rinsing test is maintained are not highly infrequent. In addition to the functional causes such as punctal malposition, eyelid laxity or lacrimal pump dysfunction, this may result from a partial narrowing of the lacrimal pathway [8]. Due to the fact that endoscopic medial maxillectomy does not affect the lacrimal pump mechanism or the external structures of the lacrimal drainage system, the eye tearing sensation reported by the patient with a positive flushing test may indicate a partial narrowing of the nasal opening. The newly formed ostium of the lacrimal pathway in patients after medial maxillectomy is located at the level of the orbital floor, usually near the anterior maxillary wall, regardless of the anatomical variant of the course of nasolacrimal duct [9]. Although the sharp transection of the nasolacrimal duct should not promote secondary scarring [10], many authors suggest using various additional techniques, such as marsupialization and flap creation [11], transcanalicullar stenting, distal stenting, or concurrent dacryocystorhinostomy (DCR) procedure to reduce the risk of post-operative eye tearing [12,13]. This, however, does not seem necessary considering the result of 4.8% of patients with eye tearing in our study group, which does not differ from previous reports. The frequency of epiphora after endoscopic maxillectomy, often combined with additional procedures aimed at improving tear patency, vary between 0% and 11.1% [10,14,15,16,17].

**Figure 3:**
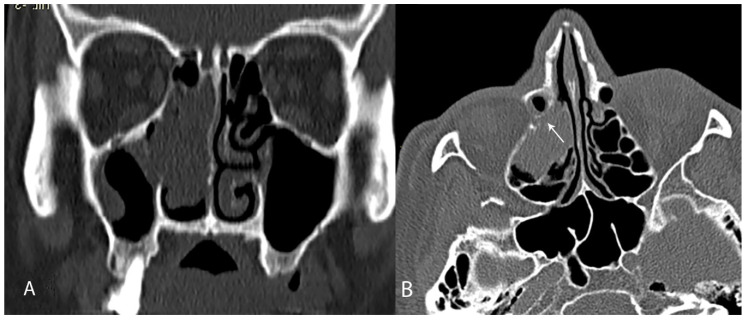
Inverted papilloma of the maxillary sinus. Tumor present in the immediate vicinity of the nasolacrimal duct—arrow, frontal plane (**A**), axial plane (**B**).

**Figure 4:**
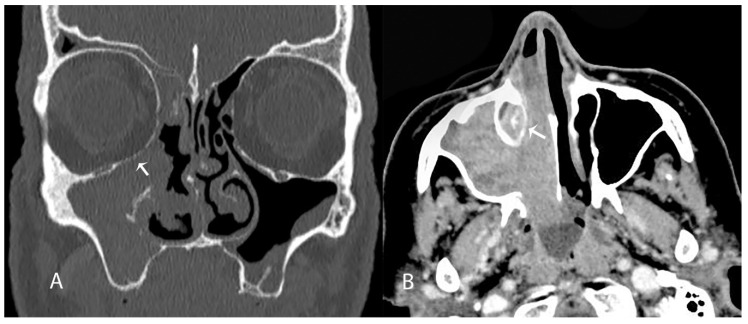
Carcinoma of the maxillary sinus and the lateral nasal cavity wall. Erosions of the lower orbital wall—arrow, frontal plane (**A**); apparent invasion of the nasolacrimal duct—arrow, axial plane (**B**).

It can be assumed that the more surgical trauma to the lower part of the lacrimal sac as a result of extensive drilling or coagulation, the greater the risk of postoperative stenosis. Consequently, malignant or locally aggressive tumors that require more aggressive surgery in this area may be associated with poorer lacrimal duct patency outcomes (Figure 3 and Figure 4). Due to the small number of malignant tumors in our study group, we do not dare to draw conclusions as to the impact of the type of tumor on the clinical outcome of surgery.

It should be noted that the methods used to assess epiphora in the cited studies are often inaccurate and are usually based on observation of clinical symptoms and patient reports [18,19]. Many authors do not provide a specific method of assessing the patency of the lacrimal ducts [14,15,16].

A number of patients with mild epiphora or sticky eyelids in the morning do not associate the symptoms with lacrimal duct obstruction or attach little significance to them. Sole reliance on a simple clinical evaluation based on patient complaints and possibly forceful irrigation of lacrimal ducts can result in a certain degree of underestimation of the number of patients with lacrimal duct obstruction after maxillectomy procedures.

Munk score is widely used to assess tear drainage disorders. Although it is a subjective method, according to some authors it allows relatively easy identification of patients with minor ailments. In our study, Munk epiphora grading indicated tear drainage disorders in 3 (14.3%) patients—three more than in the rinse test and two more than indicated by the initial clinical assessment based on the patients’ subjective reports. In both cases, epiphora was very mild and required wiping less than twice a day (Munk grade 1). Singificantly, a number of authors consider this to be a good result (operative success) in post-DCR patients [20,21].

On the other hand, patency assessment based solely on Munk score can be misleading. Sipkowa et al. [22], in post-operative assessment of patients after DCR without evident epiphora (0—Munk score), demonstrated very divergent Glasgow Benefit Inventory (GBI) scores. There is no consensus on the use of more detailed questionnaires because minor symptoms in some patients may not have a negative impact on their assessment of general or social status [22,23].

Thus, in order to increase the likelihood of detecting subtle functional disorders in our study, a modified Jones I test with direct endoscopic observation was used to supplement Munk score. Due to the ease of implementation and little discomfort for patients, the test can be performed during postoperative follow up visits. The use of a low-invasive and fast fluorescein dye disappearance test (FDDT) in some studies to assess the decrease in the amount of pigment in the conjunctival fornix necessitates the participation of a specialist (ophthalmologist) and requires additional equipment [24]. Moreover, in the case of minor patency or functional disorders, the accuracy of this test may be insufficient [25]. Consequently, some researchers suggest combining the FDDT with the Schirmer tear test strip method, in which paper strips (Clement Clarke Int., Essex, UK) were placed in the lower conjunctival fornix. However, in this context, the test does not seem to be bothersome for the patient any more.

In our study group assessed with the modified Jones I test, an increase in both drainage time by over 3 min and leakage intensity in four (19%) patients (including three patients previously identified using Munk score) were observed.

Gradually increasing the sensitivity of the method resulted in a growing number of patients with potential disorders of tear drainage, starting from 0% in the rinsing test, 4.8% self-reported tearing complaints, 14.3% Munk score, and 19% modified Jones test.

It can be suspected that the mechanism responsible for this is scar stenosis at the amputated nasolacrimal duct with retained patency. Hence, the positive effect of forceful irrigation on the one hand and the prolonged time in Jones testing with additional less intense dye leakage in the nasal passage on the other. At the same time, the outflow disturbance was not bothersome enough for patients to spontaneously report epiphora in the post-operative assessment. Patients diagnosed with functional disorders of tears outflow during careful history taking may report watery eye sensation or slight tearing, especially when the ambient temperature suddenly changed or during exposure to the wind. However, they usually did not consider these symptoms particularly troublesome and did not report them as epiphora spontaneously in the initial stage of the study. They classified this type of discomfort as a Munk score grade 1 or grade 0 and only one of our patients considered it as grade 2 epiphora.

The lack of a thorough preoperative lacrimal duct patency assessment may be considered a drawback of our research. However, some tests, especially those requiring endoscopic observation of fluorescein leakage, may be difficult to perform in the presence of the tumor filling the nasal cavity. 

Additionally, in at least some of the patients, preoperative CT examination revealed tumor invasion of the nasolacrimal canal, which of course could have had a negative effect on tear drainage in these patients. This limitation is partially compensated by the fact that the nasolacrimal duct has always been completely resected with the tumor and a healthy looking tissue margin at the level of the patent lacrimal sac. Therefore, it can be assumed that any tear drainage disturbances in the follow-up period are related to the healing process and fibrosis of the newly formed ostium of the tear duct.

## 5. Conclusions

The study results revealed that a small fraction of patients tend to report epiphora as a consequence of medial maxillectomy themselves. However, the actual proportion of drainage disorders is higher. Subtle functional disorders, which are not particularly bothersome to patients and thus are commonly neglected by them, are more common. More sensitive lacrimal duct patency tests reveal more cases of tear drainage disorders. The results of studies assessing the incidence of epiphora after medial maxillectomy appear to depend largely on the type of test used.

Similarly, little impact on general social health and disability should be expected. It can be speculated that minor drainage disorders can be noticed, felt and assessed differently by various individuals working in different conditions or performing different jobs. It can be surmised that outdoor work results in more severe epiphora, while watering of the eyes and blurred vision can be more bothersome to drivers or people performing tasks, which require precision. Further in-depth research to investigate these issues is needed.

## Data Availability

The data presented in this study are available on request from the corresponding author.
